# Emerging Link between Tsc1 and FNIP Co-Chaperones of Hsp90 and Cancer

**DOI:** 10.3390/biom12070928

**Published:** 2022-07-01

**Authors:** Sarah J. Backe, Rebecca A. Sager, Katherine A. Meluni, Mark R. Woodford, Dimitra Bourboulia, Mehdi Mollapour

**Affiliations:** 1 Department of Urology, SUNY Upstate Medical University, Syracuse, NY 13210, USA; backes@upstate.edu (S.J.B.); sagerr@upstate.edu (R.A.S.); kam445@cornell.edu (K.A.M.); woodform@upstate.edu (M.R.W.); bourmpod@upstate.edu (D.B.); 2 Upstate Cancer Center, Upstate University Hospital, Syracuse, NY 13210, USA; 3 Department of Biochemistry and Molecular Biology, SUNY Upstate Medical University, Syracuse, NY 13210, USA

**Keywords:** tuberous sclerosis complex (TSC), Tsc1 (hamartin), Tsc2 (tuberin), heat shock protein 90 (Hsp90), FNIP1, FNIP2, co-chaperones, cancer, renal cell carcinoma, kidney cancer

## Abstract

Heat shock protein-90 (Hsp90) is an ATP-dependent molecular chaperone that is tightly regulated by a group of proteins termed co-chaperones. This chaperone system is essential for the stabilization and activation of many key signaling proteins. Recent identification of the co-chaperones FNIP1, FNIP2, and Tsc1 has broadened the spectrum of Hsp90 regulators. These new co-chaperones mediate the stability of critical tumor suppressors FLCN and Tsc2 as well as the various classes of Hsp90 kinase and non-kinase clients. Many early observations of the roles of FNIP1, FNIP2, and Tsc1 suggested functions independent of FLCN and Tsc2 but have not been fully delineated. Given the broad cellular impact of Hsp90-dependent signaling, it is possible to explain the cellular activities of these new co-chaperones by their influence on Hsp90 function. Here, we review the literature on FNIP1, FNIP2, and Tsc1 as co-chaperones and discuss the potential downstream impact of this regulation on normal cellular function and in human diseases.

## 1. Introduction

Heat shock protein-90 (Hsp90) is a molecular chaperone essential for maintaining signaling competence in eukaryotic cells. Hsp90 is comprised of an N-terminal ATP binding domain, a middle domain for binding “client” proteins, and a site of constitutive dimerization at the carboxy-terminus [1,2,3]. Hsp90 function is coupled to its ability to bind and hydrolyze ATP and undergo a series of conformational changes known as the “chaperone cycle” [4,5]. This cycle facilitates the maturation and activation of more than 300-client proteins, including kinases, and non-kinases such as steroid hormone receptors, transcription factors, and tumor suppressors [6] (https://www.picard.ch/downloads/Hsp90interactors.pdf, accessed on 12 February 2022). A number of these Hsp90 client proteins participate in oncogenesis, and this chaperone machine is often co-opted by cancers to maintain deregulated signaling pathways and buffer the effect of pathogenic mutations [7,8,9,10,11]. The breadth of signaling pathways mediated by its clients makes Hsp90 an attractive therapeutic target and dozens of Hsp90-directed small molecules have been developed. In fact, there are 14-ATP-competitive Hsp90 inhibitors in ongoing clinical trials in various cancers (www.clinicaltrials.gov, accessed on 1 May 2022) [12].

The binding and dissociation of Hsp90-modulating proteins, termed co-chaperones, tailors Hsp90 to particular clients and provides directionality to the chaperone cycle [13,14,15,16]. To date, more than 25 Hsp90 co-chaperones with varying characteristics and classifications have been identified. Prior to the recent characterization of the three large co-chaperones Tsc1, FNIP1, and FNIP2 (hereon referred to as FNIP1/2), known Hsp90 regulatory proteins existed within the range of 20–100 kDa [15]. These three large co-chaperones are each approximately 130 kDa [17,18,19,20,21] and were originally identified as stabilizers of specific tumor suppressor proteins implicated in the mTOR pathway [17,18,19]. The co-chaperone function of FNIP1/2 and Tsc1 gives us an opportunity to reevaluate the previous published work from a new perspective. Here we review the functions and roles of FNIP1/2, and Tsc1 that have been reported, describe their functions as new co-chaperones of Hsp90, and retrospectively evaluate how new functions can help contextualize previous observations. We also review their roles in cancer and cellular response to Hsp90 inhibitors as well as their emerging role in chaperoning of tumor suppressors.

## 2. FNIP1 and FNIP2

### 2.1. FNIP1/2 Structure and Function

Folliculin interacting proteins 1 and 2 (FNIP1/2) are named after their first identification in complex with the tumor suppressor folliculin (FLCN) [17,18]. Loss of FLCN function is implicated in Birt-Hogg-Dubé (BHD) syndrome, a hereditary condition characterized by benign fibrofolliculomas, pulmonary cysts, spontaneous pneumothorax, and renal tumors, which are most often of hybrid oncocytic or chromophobe histology [22]. FLCN interacts with FNIP1/2 via its C-terminus, which stabilizes the FLCN protein. This mechanism is supported by the instability of C-terminally truncated FLCN protein products resulting from FLCN mutations identified in BHD [17,18,22,23] and indeed, many of these mutated FLCN proteins fail to associate with FNIPs and are targeted for proteasomal degradation [24]. Recently, portions of the FLCN:FNIP2 structure have been resolved by cryo-EM [25,26]. The structures support previous evidence that FLCN contains a GTPase activating protein (GAP) domain and interacts with FNIP2 through its C-terminal differentially expressed in normal and neoplastic cells (DENN) domain. Additionally, the N-terminal Longin domains of FLCN and FNIP2 proteins also interact, emphasizing the complex nature of the interaction between FLCN and the FNIPs [25,26]. Despite this well-supported finding, the precise mechanism by which FLCN stability is achieved had remained elusive. Our group demonstrated that FLCN is a client of Hsp90 and depends on the co-chaperone activity of FNIP1 and FNIP2 for loading to Hsp90 and thus stability [20].

**Figure 1:**
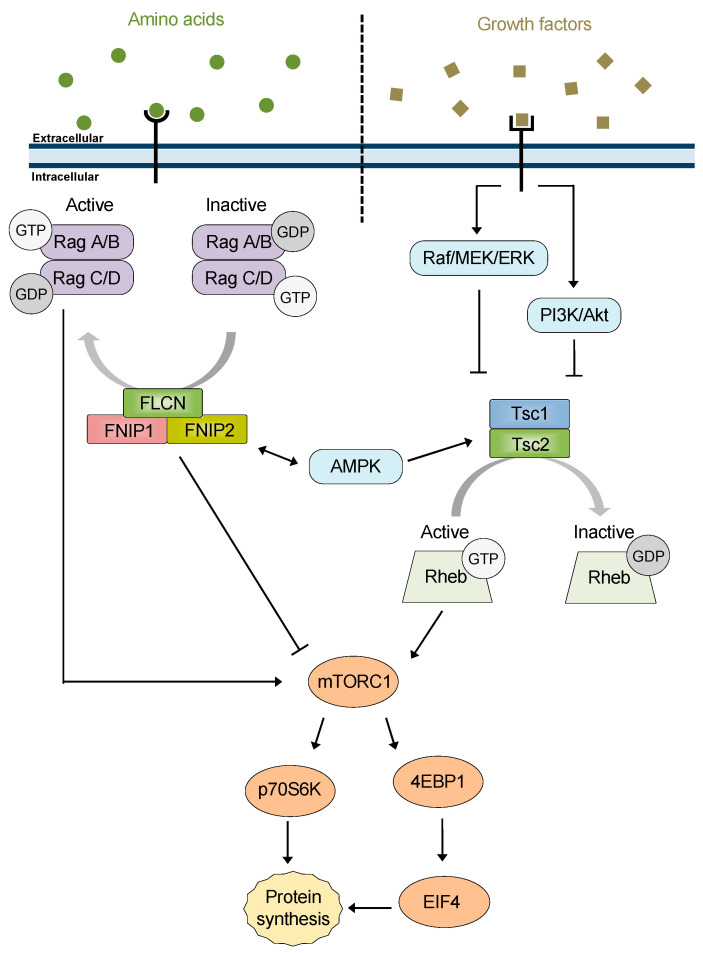
**FNIPs and Tsc1 in the mTOR pathway.** The mTOR pathway is a cellular signaling hub that integrates signals from several pathways and controls protein synthesis. A simplified schematic representation is shown to highlight the role of the FLCN/FNIPs and TSC complexes as mTOR regulators through GAP activity of RagA/C and Rheb, respectively.

FNIP1 shares 74% similarity and 49% identity with FNIP2 [18], and the majority of research on FNIPs is exclusive to FNIP1. Initially, FNIP1 was found to be phosphorylated by AMP-activated protein kinase (AMPK) as well as facilitate AMPK-mediated phosphorylation of FLCN [17]. AMPK is a negative regulator of the mTOR nutrient-sensing pathway, and FNIP1 was found to translocate from the cytoplasm to lysosomes under starvation conditions [27], therefore a role for FNIP1 in mTOR signaling was suggested, though direct, mechanistic evidence remains elusive (Figure 1).

### 2.2. FNIP1 Function in Skeletal Muscle and Adipocytes

One pathway in which the FNIP1-AMPK interaction has been interrogated is skeletal muscle fiber type specification. Broadly, type I muscle fibers are highly aerobic, express elevated levels of myoglobin, and have high mitochondrial function, while type II muscle fibers are comparatively lower in both and favor anaerobic glycolysis [28]. AMPK is known to regulate mitochondrial biogenesis via peroxisome proliferator-activated receptor-γ coactivator-1 α and β (PGC1α/PGC1β) and is activated under low energy conditions to suppress mTOR-dependent ATP utilization [29]. *FNIP1*^−/−^ mice contain an abundance of type I muscle fibers, similar to mice with gain-of-function mutations in AMPK [30,31]. This suggests that at steady state FNIP1 suppresses AMPK and thus regulates mitochondrial biogenesis. Liu et al. furthered this line of inquiry by demonstrating that miR-499, an intron of the gene encoding the major slow-twitch type I myosin heavy chain (Myh7b), directly targets and inhibits translation of FNIP1 but not FNIP2 [32]. Similar results have been shown for miR-208b [33]. Interestingly, FNIP1-mediated AMPK inhibition can be reversed by the flavonoid dihydromyricetin, which causes a decrease in FNIP1 expression and reactivates AMPK-mediated mitochondrial biogenesis [34]. These data provide a mechanism that explains the FNIP1-dependent regulation of AMPK in skeletal muscle.

FNIP1 regulation of AMPK may be cell-type dependent however, as recent work has demonstrated that FNIP1 regulates cellular respiration in adipocytes in an AMPK/mTOR independent manner [35]. Specifically, FNIP1 was shown to regulate intracellular Ca^2+^ levels through stabilization of sarcoendoplasmic reticulum calcium transport ATPase (SERCA) and increasing SERCA Ca^2+^ pump activity. This study also suggested a pivotal role for FNIP1 in regulating metabolism and glucose homeostasis in adipocytes, independent of AMPK/mTOR [35].

### 2.3. FNIP1 in Oxidative Stress

An interesting perspective on FNIP1 regulation of AMPK activity can be gained through an understanding of the factors governing FNIP1 protein dynamics. Recent work has shown that reductive stress promoted the degradation of FNIP1, but not FNIP2 [36]. The mechanism was traced to the chelation of Zn^2+^ by two reduced Cys residues in FNIP1, which recruits CUL2^FEM1B^ [36,37], the scaffold and recognition subunits of an E3-ubiquitin ligase complex [38]. Degradation of FNIP1 in this context promotes AMPK-PGC1α-mediated mitochondrial biogenesis to counteract reductive stress [36,37]. Interestingly, loss of FEM1B led to decreased lactate production [36], perhaps as a byproduct of FNIP1-dependent stabilization of FLCN and its recently described tumor suppressive effect on lactate dehydrogenase A [39].

### 2.4. FNIP1 Function in B-Cell Development

Another striking example of FNIP1 function is in lymphoid differentiation and maturation. Park et al. identified a pre-B cell “checkpoint” where loss of FNIP1 prevents mature B-cell development [40]. These cells were found to be sensitive to nutrient-deprivation-induced apoptosis seemingly due to failure of AMPK to suppress mTOR in the absence of FNIP1 [40]. Interestingly, FNIP1-deficient B-cell progenitors also exhibit elevated TFE3 transcription as well as increased lysosome function and autophagic flux [41]. Similarly, loss of FNIP1 prevents maturation of invariant natural killer T cells and increases their sensitivity to apoptosis [42]. *FNIP1* knockout was again determined to cause downstream mTOR activation, though in this case the effect is definitively indirect, as rapamycin treatment was not able to rescue the phenotype [42]. Concurrent research also found a marked pre-B cell blockade and confirmed that the effect stems from caspase activation and intrinsic apoptosis [43]. This effect was also observed in patients, as *FNIP1* mutation caused a clinically significant reduction in B cell numbers and hypogammaglobulinemia [44,45]. In addition to B-cell deficiency, FNIP1 loss leads to cardiomyopathy, which phenocopies AMPK gain-of-function mutations, consistent with a failure of FNIP1 to regulate AMPK-mediated signaling [46]. Taken together, these data support a role for FNIP1 as an indirect regulator of mTOR through its suppression of AMPK activity, and likely also via its positive regulation of FLCN [47].

### 2.5. Role of FNIP1/2 in Transcription

Recent work has also demonstrated the impact of the FLCN-FNIP1/2 system on transcriptional reprogramming. It is well established that loss of FLCN induces nuclear localization of the transcription factors TFE3/TFEB and promotes a gene expression program favorable for tumor growth [41,48,49,50,51]. Similarly, it was recently shown that simultaneous deletion of *FNIP1/2* in a human renal proximal tubular epithelial cell (RPTEC) line induced a TFE3-mediated gene signature [52]. This is in agreement with previous data showing that *FLCN*-null and *FNIP1/2*-null mice developed phenotypically indistinguishable enlarged polycystic kidneys [53,54]. Additionally, loss of either *FLCN* or *FNIP1/2* induced a STAT2-dependent interferon response transcriptional program, though the impact of interferon signaling in *FLCN*-deficient tumors is unclear [52].

Despite the progress reviewed here, it remains difficult to disentangle the cellular roles of FNIP1/2 in the regulation of AMPK and TFE3 from that of FLCN tumor suppressive function. Given this, it is possible that FNIP1/2-mediated regulation of Hsp90 activity provides a unifying explanation for FNIP-mediated cellular effects.

## 3. Tsc1

### 3.1. Structure and Function of the Tsc1/2 Complex

Tuberous Sclerosis Complex (TSC) is an autosomal dominant genetic syndrome caused by mutations in either the *TSC1* or *TSC2* tumor suppressors. In addition to neural associations that include epilepsy, subependymal giant cell astrocytomas (SEGA), intellectual disability, and autism, TSC is also characterized by cutaneous, pulmonary, and renal manifestations, similar to BHD [23,55,56]. These include facial fibrofolliculomas, pulmonary lymphangiomyomatosis, and renal angiomyolipomas (AML). The *TSC2* gene was cloned first in 1993 followed by the non-homologous *TSC1* gene in 1997 [19,57]. The Tsc1 and Tsc2 proteins, also known as hamartin and tuberin, respectively, were then shown to directly interact and form a complex [58]. The 130 kDa Tsc1 and 200 kDa Tsc2 proteins share no homology with each other [59]. Recently, partial structures of this complex were resolved by cryo-EM and revealed an elongated structure with a 2:2 stoichiometry. Further, Tsc1 was consistently found to have a coiled-coil domain which mediated Tsc1 dimerization and interaction with Tsc2 in vitro [60,61,62]. This is in contrast to a previous study using a yeast two-hybrid system which identified Tsc1 residues 302–430 as the critical region for Tsc2 interaction [63]. Tsc2 interaction with Tsc1 was primarily mediated through the N-terminal Tsc2-HEAT repeat domain, which is consistent with previous findings [60,62,63]. Furthermore, Tsc1 was required for Tsc2 maximal GAP activity likely through proper positioning of the Tsc2 catalytic asparagine-thumb [62].

The Tsc1/2 complex was demonstrated to inhibit mTOR signaling through the GAP activity of Tsc2 towards Rheb [64,65,66] (Figure 1). The effect of Tsc2 was greatly potentiated by the presence of Tsc1. In this TSC complex, Tsc1 has been shown to be important for the stabilization of Tsc2, preventing its interaction with the HERC1 ubiquitin ligase and its ubiquitination [67,68]. Early identification of Tsc1 and Tsc2 in complex and the role of this complex in the mTOR pathway focused a large portion of the TSC literature on this function and does not address separable functions of Tsc1 and Tsc2.

### 3.2. Separable Functions of Tsc1 and Tsc2

There are a number of differences in Tsc1 and Tsc2 function that have been identified, as well as mTOR-independent functions. Early reports suggested that although Tsc1 and Tsc2 often co-localize, the subcellular locations as well as tissue and organ expression patterns of Tsc1 and Tsc2 are not identical [69]. Germline mutations in *TSC1* cause a similar but not identical phenotype to *TSC2* mutations in animal models, suggesting commonalities to the pathways involved but some differences as well [70]. Renal tumors developed in heterozygous *TSC1* mice at a slower rate than in *TSC2*^+/−^ mice. In addition to renal cystadenomas, *TSC1*^+/−^ mice also develop liver hemangiomas, which are more common and more severe in female mice, demonstrating sex-dependent lethality [71]. Concordantly, an analysis of patients in the TOSCA database (TuberOus SClerosis registry to increase disease Awareness) revealed that female patients were significantly more likely to develop renal AML and experience hemorrhage [72]. Sex-dependent and estrogen linked effects exclusive to Tsc1 can also be seen in mammary development. Conditional Tsc1 loss in mammary epithelium impaired mammary development through suppression of Akt, ER, and cell cycle regulators and did not lead to tissue hyperplasia [73]. Moderate overexpression of Tsc1 also enhances overall health and cardiovascular health in an animal model and improves survival only in female mice [74]. Tsc1 and Tsc2 have also been shown to have separable functions in both cell signaling and cell cycle control [75,76]. Milolaza et al. describe the effect of Tsc1 and Tsc2 on the G1 to S phase transition of the cell cycle. Tsc1 and Tsc2 control cell proliferation independent of each other, and only Tsc2 function is affected by p27 expression [75]. Further evidence for separate functions of Tsc1 and Tsc2 comes from microarray and proteomic approaches, which reveal that each TSC gene triggers substantially different cellular responses [77,78,79,80,81,82].

### 3.3. mTOR Independent Functions of Tsc1

While the effects of Tsc1 loss are often ascribed to increased mTOR signaling and are at least partially responsive to rapamycin, there are also mTOR independent functions of Tsc1 that have been reported. Tsc1 haploinsufficiency without mTOR activation was shown to lead to renal cyst formation in *TSC1*^+/−^ mice [83]. It has also recently been demonstrated that p21 activated kinase 2 (PAK2) is an effector of the Tsc1/Tsc2 complex. Loss of either Tsc1 or Tsc2 promotes hyperactivity of PAK2 downstream of Rheb, but independent of mTOR, as demonstrated by insensitivity to rapamycin treatment [84]. Tsc1 and Tsc2 also differentially modulate the cytoskeleton. *TSC1*^−/−^ and *TSC2*^−/−^ MEFs demonstrate rapamycin insensitive increase in number and length of cilia [85] whereas only Tsc2 loss promotes an mTOR-dependent pro-migratory phenotype [86]. On the other hand, Tsc1 loss was shown to dysregulate tight junction development in an mTOR independent manner [87]. Collectively, these studies suggest a role for Tsc1 in cell integrity independent of mTOR.

Furthermore, it has long been observed that clinical features of TSC across multiple organ systems are more severe in patients with mutations in *TSC2* than in patients with *TSC1* mutations [88,89,90]. There is a higher incidence of intellectual disability in patients with *TSC2* mutations, and it has been suggested that severity of disability may correlate with predicted effects of mutations on Tsc1 and Tsc2 protein [91,92,93]. Epilepsy generally exhibits an earlier onset and is also more severe as a result of *TSC2* mutations [94,95]. Similarly, the mean age at diagnosis for patients with renal AML was lower in patients with *TSC2* mutations. Additionally, patients with *TSC2* mutations had a higher occurrence of renal AML, multiple renal cysts and polycystic kidney disease compared to patients with *TSC1* mutations [72]. In a mouse model, conditional knockout (CKO) of *TSC2* in GFAP-positive cells also produces a more severe epilepsy phenotype than *TSC1* CKO [96]. Additionally, it has been proposed that perhaps TSC resulting from *TSC1* mutation is not less common than *TSC2* disease but that it is less frequently diagnosed due to the milder clinical features [97].

Collectively, this evidence suggests a role for Tsc1 outside the TSC complex and mTOR signaling. Due to the described role of Tsc1 in stabilizing Tsc2 and protecting it from ubiquitination we questioned whether this protective role involved molecular chaperones and whether Tsc1 was involved in chaperoning Tsc2. In fact, Tsc2 is a client of Hsp90, and Tsc1 is a co-chaperone [21].

## 4. Regulation of Hsp90 Chaperone Function by Co-Chaperones

**Figure 2:**
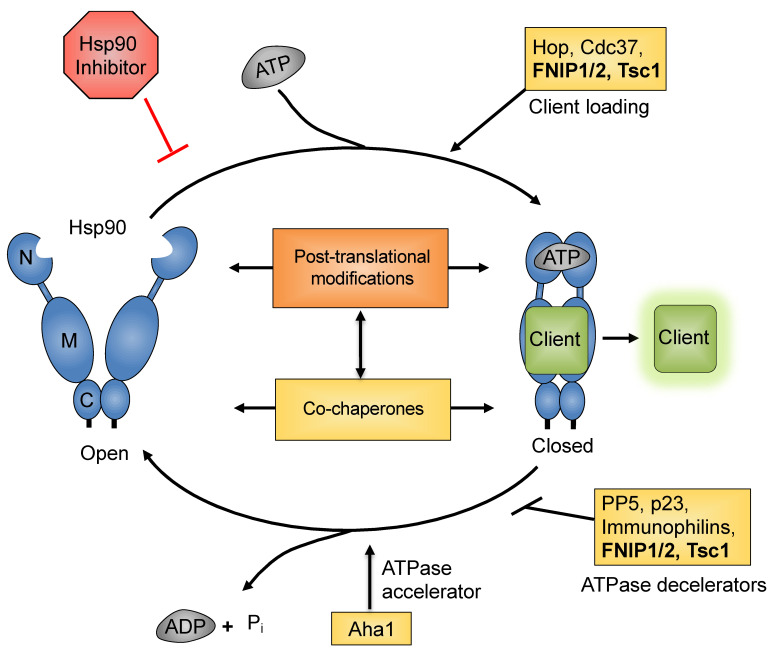
**The Hsp90 chaperone cycle.** Open Hsp90 is dimerized only through contacts in the CTD. ATP binding and an ordered series of conformational changes allow Hsp90 to adopt a closed conformation, which is N-terminally dimerized. ATP hydrolysis leads Hsp90 to return to the open conformation and begins another chaperone cycle. Throughout the chaperone cycle co-chaperones bind to Hsp90 and regulate its function. PTM of Hsp90 and PTM of co-chaperones provide further regulation of the chaperone cycle.

The action of co-chaperones towards Hsp90 generally meets one or more of the following criteria: (1) scaffolding of client proteins to Hsp90 (e.g., Hop, p50^Cdc37^); (2) modulation of Hsp90 ATPase activity (e.g., Aha1); (3) stabilization of specific chaperone complexes (e.g., p23) and are not themselves dependent on Hsp90 for stability [98]. We have shown that the newly identified large co-chaperones FNIP1/2 and Tsc1 are able to satisfy at least two of these observed co-chaperone functions (Figure 2). Indeed, we have a unique opportunity to advance our understanding of the function and effect of these proteins as we reconcile their known functions with their roles as Hsp90 co-chaperones.

Hsp90-dependent maturation and activation of client proteins relies on a continuum of regulated conformational changes of Hsp90 coupled to ATP hydrolysis. As currently understood, there are several “stages” to a generalized chaperone cycle. Initially, immature clients bind to the early chaperone heat shock protein 70 (Hsp70) and the Hsp70-Hsp90 organizing protein (Hop) forms a bridge to the “open” conformation of Hsp90, allowing the transfer of a client protein to Hsp90 [99]. ATP subsequently binds to the amino-terminal nucleotide binding pocket, and concurrent binding of the Activator of Hsp90 ATPase (Aha1) displaces Hop and induces transient N-domain dimerization, forming the “closed 1” state. Aha1 binds to the N-domain as well as the middle domain of Hsp90 and greatly increases the weak intrinsic ATPase activity of Hsp90 [100]. Interaction of the co-chaperone p23 with the N-domain of Hsp90 displaces Aha1 and stabilizes the “closed and twisted” conformation (closed 2). This allows completion of ATP hydrolysis, followed by release of a mature client protein and the return of Hsp90 to the open conformation [101,102,103].

The complement of co-chaperones that regulate Hsp90 during a single chaperone cycle is largely dependent on the individual requirements of the client protein [104]. For example, kinase clients are loaded to Hsp90 by the co-chaperone Cdc37, a decelerator of Hsp90 ATPase activity, and protein phosphatase 5 (PP5)-mediated dephosphorylation of Cdc37 is required for their release [105,106]. Alternatively, overexpression of Aha1 greatly decreases the folding of CFTR by accelerating the rate of Hsp90 ATP hydrolysis [107,108,109]. Similarly, steroid hormone receptors prefer a slower chaperone cycle and require the co-chaperone p23, which is known to decelerate the action of Hsp90 [5,110,111,112]. In fact, GR itself is capable of modulating the conformation of Hsp90 such that Hsp90 ATPase activity decreases [113], demonstrating the degree of specificity that can be achieved by modulation of Hsp90 complexes [15].

## 5. FNIP1/2 and Tsc1: New Co-Chaperones of Hsp90

**Figure 3:**
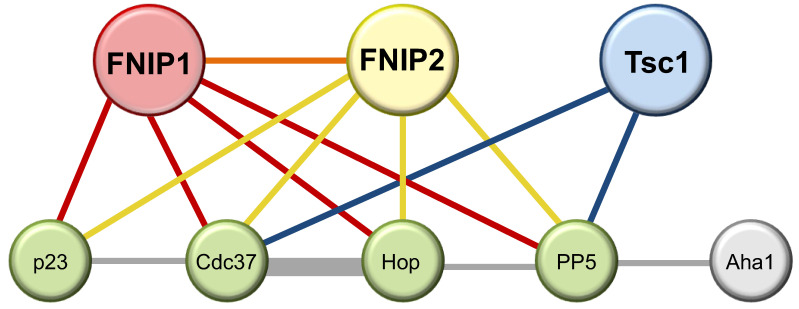
**FNIPs and Tsc1 co-chaperone interaction network.** Hsp90 co-chaperones are represented by colored circles. Interactions between co-chaperones are denoted by colored lines. FNIP1 interactions are colored red; FNIP2, yellow; Tsc1, blue; other, gray.

Recent reports from Mollapour’s group demonstrated that the tumor suppressors FLCN and Tsc2 are Hsp90 clients [20,21]. As FNIP1/2 and Tsc1, respectively, have established roles as guardians of these tumor suppressors [17,18,67,68], it follows that there may be a role for molecular chaperones in mediating FLCN and Tsc2 stability. Indeed FNIP1/2 and Tsc1 both interact with Hsp90 and Hsp70, as well as with overlapping complements of Hsp90 co-chaperones including PP5, Cdc37, Hop, and p23 and behave as Hsp90 co-chaperones [20,21] (Figure 3). These reports also demonstrate a role for these new co-chaperones in regulating both kinase and non-kinase clients, as well as provide clues to their chronology in the overall chaperone cycle.

FNIP1 and Tsc1 share a number of striking similarities in their actions as co-chaperones. Both FNIP1 and Tsc1 exhibit complex binding to Hsp90; contacts are made using multiple domains of these co-chaperones as well as multiple domains of Hsp90. The most well characterized interactions thus far however are that of FNIP1 and Tsc1 binding the Hsp90 middle domain via their carboxy-termini. It is through this interaction that they decelerate Hsp90 ATPase activity and compete with the accelerating co-chaperone Aha1 for Hsp90 occupancy. In addition to increasing the dwell time of ATP (and thus client proteins) on Hsp90, interaction with these large co-chaperones also increases Hsp90 binding to its ATP-competitive inhibitors [20,21,114].

While the overall pattern of how FNIP1 and Tsc1 interact with Hsp90 is similar there are key differences between them. The C-terminal fragment of Tsc1 (Tsc1-D) binds to Hsp90 with higher affinity than does the C-terminal fragment of FNIP1 (FNIP1-D). Similarly, Tsc1-D is a potent inhibitor of Hsp90 ATPase activity and very effectively competes with Aha1 for Hsp90 binding as evidenced by in vitro competition experiments. FNIP1 and Tsc1 can also be distinguished by the complement of co-chaperones with which they interact therefore, providing clues to their distinct roles in the chaperone cycle. While neither is found in complex with Aha1, Tsc1 is able to interact with PP5 and Cdc37, whereas FNIP1/2 can additionally be found in complexes containing p23 and Hop (Figure 3). This may demonstrate some promiscuity of FNIPs, but likely reflects the necessity for FNIPs to work in concert with other co-chaperones, while Tsc1 may be capable of modulating Hsp90 independently. This potentially explains the observation that Tsc1 is a much more potent decelerator of Hsp90 ATP hydrolysis than FNIP1/2 [20,21,114]. Interestingly, Tsc1 also inhibits the ATPase activity of another molecular chaperone, Hsp70, in vitro [115]. Whether FNIPs share this function remains unknown.

Despite their shared role in facilitating chaperoning of both kinase and non-kinase clients, FNIP1/2 and Tsc1 over-expression and deletion have different effects. Non-kinase clients are destabilized upon knockdown/knockout of FNIP1/2 or Tsc1 and stabilized with overexpression of the co-chaperones. Interestingly, FNIP1/2 knockdown or overexpression affects the kinase clients in a comparable manner as the non-kinase clients, however overexpression or absence of Tsc1 both negatively affect kinase client stability [20,21]. This may be due to the participation of FNIP1/2 with a variety of chaperone complexes, whereas the semi-exclusive nature of Tsc1 co-chaperone activity disrupts the delicate balance of Hsp90 co-chaperone dynamics.

### 5.1. FNIP1/2 and Tsc1 in the Chaperone Cycle

**Figure 4:**
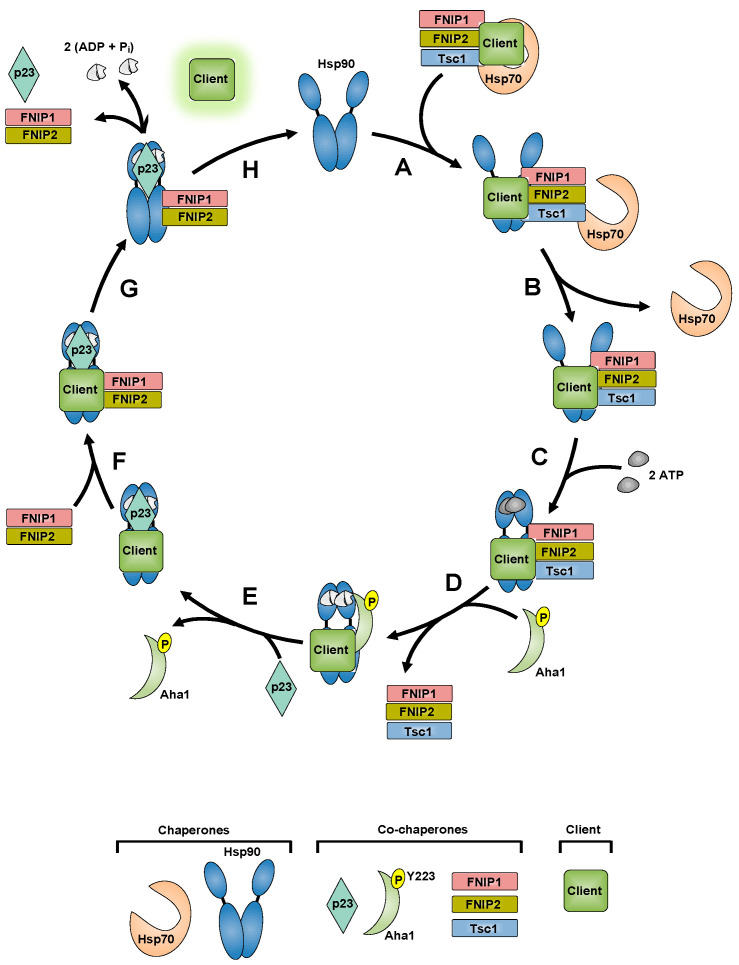
**FNIPs and Tsc1 in the Hsp90 chaperone cycle.** (**A**) FNIPs and Tsc1 co-chaperones scaffold a client from Hsp70 to Hsp90. (**B**) Hsp70 dissociates from the complex. (**C**) ATP binding triggers Hsp90 conformational rearrangements resulting in the ‘closed’ conformation. (**D**) Aha1 phosphorylated at Y223 displaces FNIPs/Tsc1 co-chaperones from the Hsp90 complex and promotes ATP hydrolysis to ADP + Pi. (**E**) p23 binds and stabilizes the closed conformation of Hsp90. (**F**) FNIP co-chaperones bind to the Hsp90:client:p23 complex to promote client maturation. (**G**) The complex dissociates releasing the mature client. (**H**) Hsp90 is reset to begin another cycle.

This large body of work on co-chaperone dynamics allows us to propose a model of FNIPs and Tsc1 co-chaperones in the Hsp90 chaperone cycle. Our previous work demonstrates that FNIPs and Tsc1 interact with Hsp70 in addition to Hsp90, and FNIP1 and Tsc1 are essential for scaffolding FLCN and Tsc2, respectively, to Hsp90 (Figure 4A,B) [20,21,23]. Subsequent ATP binding triggers conformational changes leading to the N-terminally dimerized ‘closed’ conformation of Hsp90 (Figure 4C) [116,117,118,119]. We have previously shown that Tsc1 and FNIP1 are not found in complex with Aha1 and that phosphorylation of Aha1-Y223 displaces Tsc1 from Hsp90 (Figure 4D) [20,21,109]. p23 is a late-acting co-chaperone that locks Hsp90 in the closed conformation to allow proper client maturation (Figure 4E) [103,120,121,122,123,124,125,126,127]. FNIP1/2, but not Tsc1, are found in complex with p23 (Figure 4F) [20,21]. We propose that p23:FNIP1:FNIP2 holds the matured client in its active conformation until there is a signal for client release, resetting Hsp90 for another cycle (Figure 4G,H).

### 5.2. FNIPs, Tsc1 and the Chaperone Code

Hsp90 and its co-chaperones’ functions are heavily regulated by post-translational modifications (PTM), collectively known as the ‘chaperone code’ [128,129]. An additional layer of Hsp90 regulation via FNIP1 is provided through FNIP1 post-translational modification. Recent work by our group identified a series of serine residues (S938, S939, S941, S946, and S948) in the Hsp90-binding region of the FNIP1 carboxy-terminus that are phosphorylated in a relay manner by casein-kinase 2 (CK2) [114]. This sequential phosphorylation promotes FNIP1 interaction with Hsp90 while dephosphorylation of these residues by the Hsp90 co-chaperone PP5 disrupts the Hsp90-FNIP1 complex. Furthermore, stepwise phosphorylation of FNIP1 provides gradual inhibition of Hsp90 ATPase activity and therefore increased activity of a subset of both kinase and non-kinase clients [114].

These new co-chaperones also affect Hsp90 binding to its ATP-competitive inhibitors. Generally, there is an inverse relationship between the rate of ATP hydrolysis and the ability of Hsp90 to bind ATP-competitive inhibitors [20,21,109]. Overexpression of FNIP1/2 or Tsc1 decreases Hsp90 ATPase activity, thus increasing Hsp90 binding to its inhibitors. As expected, Hsp90 inhibitor binding is decreased upon knockdown of FNIP1/2 or loss of Tsc1 [20,21,114,130]. Interestingly, approximately 15% of bladder cancers have loss-of-function mutations in Tsc1. Tsc1 loss causes hypo-acetylation of Hsp90 on K407 and K419 leading to decreased binding of Hsp90 to its inhibitors, demonstrating another mechanism of Tsc1-mediated regulation of Hsp90 [130]. The precise mechanism of how Tsc1 loss compromises Hsp90 acetylation remains unknown, however it is important to note that Hsp90 acetylation can be restored by histone-deacetylase (HDAC) inhibition, sensitizing *TSC1*-null cells to Hsp90 inhibitors [130].

Targeting Hsp90 in cancer cell lines induces apoptosis, and Hsp90 inhibitors have been found to preferentially accumulate in cancer cells versus normal cells [131,132,133,134,135]. FNIP1/2 were found overexpressed in cancer cell lines originating from several different tissues, and knockdown decreased sensitivity of these cancers to Hsp90 inhibition [20]. This increased expression of FNIP1/2 provides one potential mechanism to explain the tumor selectivity of Hsp90 inhibitors. Similarly, bladder cancer cells lacking functional Tsc1 fail to accumulate Hsp90 inhibitors and are less sensitive to Hsp90 inhibition than those with wild-type Tsc1 [130].

Collectively, these studies provide support for a new functional role for the tumor suppressor Tsc1 and FNIP1/2 as co-chaperones of Hsp90. As Hsp90 co-chaperones the protective function of Tsc1 and the FNIPs goes beyond mediating stability of Tsc2 and FLCN, respectively, and provides insight into a larger role for these proteins in the cellular context.

## 6. A New Perspective: FNIPs, Tsc1, and mTOR

Early connection of FNIP1/2 and Tsc1 to the mTOR nutrient-sensing pathway has narrowed the focus of research conducted on these proteins. Recent research has demonstrated that FNIP1/2 and Tsc1 act as co-chaperones of Hsp90. This allows us to reevaluate the previous published work with a new perspective.

### 6.1. FNIP1/2 Co-Chaperone Activity Contributes to mTOR Regulation

**Figure 5:**
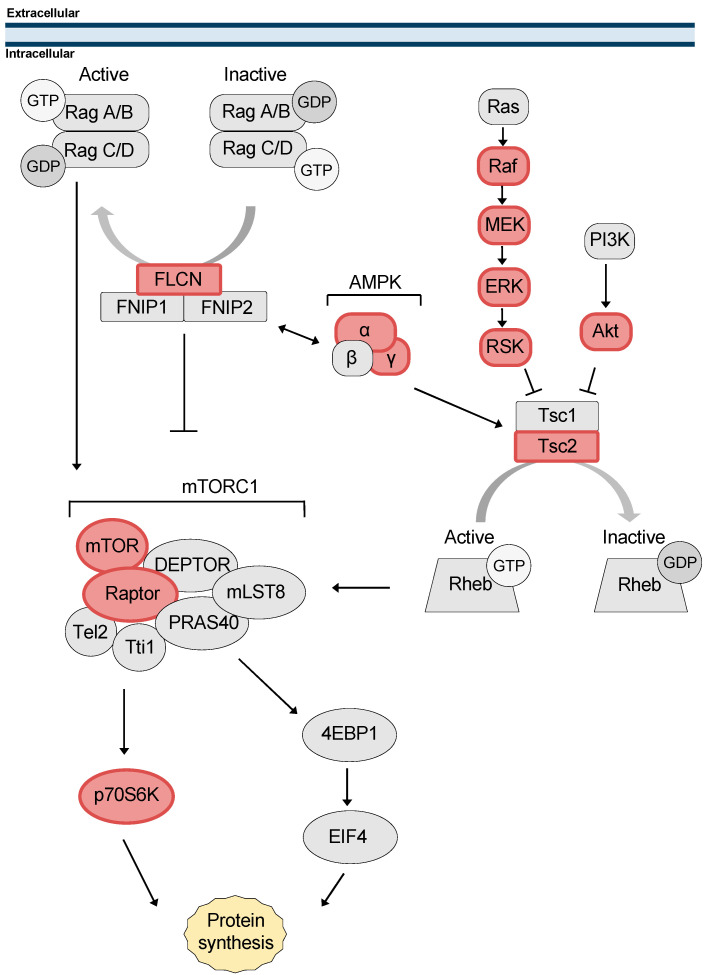
**Hsp90 clients in the mTOR pathway.** A schematic representation of the mTOR pathway highlighting the components that are Hsp90 clients (red).

As reviewed in this text, FNIP1 negatively regulates AMPK activity. Since the α and γ subunits of AMPK are known clients of Hsp90 [136], the effect of FNIP1 on AMPK could be mediated through the Hsp90 chaperone (Figure 5). In support of this idea, microarray data show that B220^+^CD43^+^ pre-B cells from *FNIP1*^−/−^ mice demonstrate a dramatic increase in expression of AMPK-responsive genes [40]. These data would suggest a role for FNIP1 in activation of mTOR, however we posit that this mechanism may actually be more complicated. First, mTOR is also an Hsp90 client protein [137] and will be subject to the influence of Hsp90 co-chaperones as with any other client protein. Second, it is likely that loss of FLCN is actually responsible for mTOR activation, as is suggested by Baba et al., whose data show that the induction of mTOR is mild in *FNIP1*^−/−^ mice as compared to *FLCN*^−/−^ and that *FNIP1* deletion fails to phenocopy BHD syndrome [17,43,54]. Concordantly, FNIP co-chaperone activity toward FLCN can explain the observation that non-degradable FNIP2 enhances FLCN expression and thus suppresses tumorigenesis in a BHD mouse xenograft model [138]. Together, these data highlight that the co-chaperone activity of FNIP1/2 is essential for FLCN-mediated mTOR suppression, but also underscore our inability to reconcile this observation with the current understanding of FNIP1/2 function.

### 6.2. Co-Chaperone Activity of Tsc1 in Regulation of mTOR

The newly identified role for Tsc1 as an Hsp90 co-chaperone may help clarify some of the phenotypic differences as a result of Tsc1 versus Tsc2 mutation. Due to its function as a co-chaperone, Tsc1 loss would trigger effects on many cellular pathways, not just mTOR signaling. This could explain the finding of renal cyst formation in *TSC1*^+/−^ mice, as well as provide insight into the pro-migratory phenotype seen only in *TSC2*^−/−^ MEFs [83,86]. Furthermore, Tsc1 loss, but not Tsc2 loss, causes hypo-acetylation of Hsp90 further demonstrating a role for Tsc1 independent of both Tsc2 and mTOR [130]. The loss of Tsc1 has a dramatic negative effect on Hsp90 kinase and non-kinase clients, including Tsc2. It is reasonable therefore that loss of Tsc1 co-chaperone activity manifests itself independently of the mTOR pathway.

As discussed above, Tsc1 loss has long been known to lead to a less severe phenotype than Tsc2 loss both in patients as well as animal models [88,89,90,94,95,96,97]. Canonically, Tsc2 loss leads to upregulation of mTOR signaling due to release of the inhibitory signal from Tsc2-Rheb [139]. Activation and phosphorylation of mTOR and its downstream targets as well as other pathway components such as Akt has been shown to be dependent on Hsp90; in fact, many mTOR pathway components are clients of Hsp90 [140,141,142,143] (Figure 5). Activation of the mTOR pathway is therefore dependent on proper function of the Hsp90 chaperone system. Perhaps the milder phenotype seen with Tsc1 loss is a result of the loss of co-chaperone activity toward Hsp90. Upon Tsc1 loss, Tsc2 is destabilized leading to increased mTOR activity; however, the other proteins in the mTOR pathway that are Hsp90 clients would also be destabilized, potentially mitigating the downstream effect.

Rapalogs, such as sirolimus and everolimus, are rapamycin derivatives that are commonly used to treat patients with BHD and TSC. Armed with the information of the role of Hsp90 in BHD and TSC, perhaps preclinical examination of Hsp90 inhibitors in combination with rapalogs is warranted. In fact, Hsp90 inhibitors have been shown to synergize with PI3K/Akt/mTOR inhibitors in preclinical studies for the treatment of various cancers [144,145,146]. In line with this, Di Nardo et al. identified the heat-shock machinery as an exploitable target in Tsc2-deficient neurons [147]. Accordingly, one study has evaluated mTOR and Hsp90 inhibitors in combination in *TSC1* or *TSC2* deficient cancer models. Unfortunately, the results were inconsistent between cell line and mouse xenograft models, as synergism between Hsp90 inhibitor (GB) and mTOR inhibitors (Torin2, rapamycin) in cell lines did not translate to increased efficacy over monotherapy in xenograft models [148]. Taken together, these studies demonstrate the potential therapeutic benefit of co-targeting Hsp90 and mTOR in BHD and TSC patients. However, further investigation is needed.

## 7. Specialized Function of FNIP1/2 and Tsc1: Chaperoning Tumor Suppressors

An important and perhaps specialized role for these new Hsp90 co-chaperones is in the chaperoning of tumor suppressors. FLCN and Tsc2 are additions to the growing list of tumor suppressors that interact with Hsp90. The transcription factor p53 was the first reported tumor suppressor client of Hsp90 [149,150,151,152]. Since 1996, 17 functional Hsp90 interactions with both kinase and non-kinase tumor suppressors have been discovered (Table 1) [20,21,149,153,154,155,156,157,158,159,160,161,162,163]. Furthermore, several tumor suppressors including, VHL, BDC2, LKB1, p53, FLCN and LATS1/2 were found to interact with Hsp90 co-chaperones including, Hop, p23, Hsp110, Cdc37, PP5, and CHIP [20,23,150,154,159,160,164,165,166,167]. As FNIP1/2 and Tsc1 scaffold the tumor suppressors FLCN and Tsc2 to Hsp90, it follows that these co-chaperones may participate in the chaperoning of additional Hsp90-dependent tumor suppressor clients. In line with this notion, Tsc1 was identified as a genetic interactor with VHL in HeLa cells highlighting the need for further exploration in this area [168].

Additionally, there is new evidence supporting a compensatory mechanism between FNIP1/2 and Tsc1 co-chaperone activity. FLCN traditionally requires interaction via its C-terminus to FNIP1/2 to mediate its stability. Tsc1, however, is capable of interacting with a truncated FLCN mutant and supporting a low level of expression in the absence of FNIP1/2 binding [23]. Unexpectedly, the truncated FLCN-L460QsX25 was still able to interact with Hsp90 even though it did not bind to its loading co-chaperone FNIP1. Overexpression of Tsc1, but not FNIP1 was capable of stabilizing expression of the mutant FLCN. Notably, Tsc1 interaction with Tsc2 was compromised in this model resulting in loss of Tsc2 tumor suppressive function. Loss of such a compensatory mechanism may also explain why deletion of *FNIP1* synergized with *TSC1* deletion to activate mTOR and subsequently resulted in accelerated renal cyst formation in mice [189]. These findings necessitate investigation into how these large co-chaperones mediate chaperoning of tumor suppressors and the impact of tumor suppressor mutations on this relationship.

## 8. Conclusions

Hsp90 is an important component of the cellular homeostatic machinery and is regulated by post-translational modification and interaction with co-chaperones. There are more than 25 known co-chaperones that serve several functions including modulating Hsp90 conformations, loading client proteins to Hsp90, and modifying the rate of ATP hydrolysis. New data has identified newly characterized roles for three proteins, FNIP1, FNIP2 and Tsc1, as large co-chaperones of Hsp90. Though these proteins have established roles in the regulation of tumor suppressor proteins FLCN and Tsc2, it seems that many of their other ascribed functions are potentially explained at least in part by their effect on Hsp90. A more thorough understanding of the action and interplay of these new, large co-chaperones may unveil clues that will aid in developing the next generation of cancer therapeutics.

## Figures and Tables

**Table 1:** Relationships of known tumor suppressor-Hsp90 interactions.

Tumor Suppressor Gene	Relationship to Hsp90	References
ATM Kinase	Client	[153,169]
BMPR1A	Client	[161]
BRCA1/2	Client	[158,170,171]
DBC2	Client	[159]
FBXW7	Interactor	[161]
FLCN	Client	[20,23]
IRF1	Client	[156]
LATS1/2	Client	[162,167,172]
LKB1	Client	[154,173,174]
NDRG2	Interactor	[163]
SYK	Client	[155,175]
TNFAIP3	Interactor	[161,176]
TP53	Client	[8,103,149,150,151,152,164,165,166,177,178,179,180,181,182,183,184,185,186,187]
TSC1	Co-chaperone	[21,130]
TSC2	Client	[21]
VHL	Client	[160,188]
WT1	Client	[157]

## Data Availability

Not applicable.
